# EHMTI-0378. Imaging sensory effects of occipital nerve stimulation: a new computer-based method in neuromodulation

**DOI:** 10.1186/1129-2377-15-S1-K4

**Published:** 2014-09-18

**Authors:** A Göbel, C Göbel, A Heinze, U Niederberger, D Rasche, HM Mehdorn, H Göbel

**Affiliations:** 1 Department of Neurology, University Hospitals Schleswig-Holstein Campus Lübeck, Kiel, Germany; 2 Department of Neurology, University Hospital Cologne Germany, Kiel, Germany; 3 Kiel Migraine and Headache Centre, Kiel Pain Centre, Kiel, Germany; 4 Institute of Medical Psychology and Medical Sociology, University Hospital of Schleswig-Holstein Campus Kiel, Kiel, Germany; 5 Department of Neurosurgery, University Hospitals Schleswig-Holstein Campus Lübeck, Lübeck, Germany; 6 Department of Neurosurgery, University Hospitals Schleswig-Holstein Campus Kiel, Kiel, Germany

## Background

Within the last years, occipital nerve stimulation has proven to be an important method in the treatment of severe therapy-resistant neurological pain disorders. The correspondence between electrode placement as well as possible stimulation parameters and the resulting stimulation effects remains unclear.

## Objective

The method aims to directly relate the neuromodulatory mechanisms with the clinical treatment results, to achieve insight in the mode of action of neuromodulation, to identify the most effective stimulation sets and to optimise individual treatment effects.

## Methods

We describe a new computer-based imaging method for mapping the spatial, cognitive and affective sensory effects of occipital nerve stimulation. The procedure allows a quantitative and qualitative analysis of the relationship between electrode positioning, the stimulation settings as well as the sensory and clinical stimulation effects

## Results

The mapping can be carried out with different given stimulation parameters, to qualitatively and quantitatively capture the effects of the chosen parameters. The patient first documents the programme, for which he or she wishes to map the treatment effects. Using the IPG control device, the stimulation intensity for the respectively used programme are selected and then documented using the graphical user interface. Thereafter, patients are able to map the sensory experiences and their intensity.

## Conclusion

The new computer based imaging procedure allows a continuous tracking of progress and success of neuromodulation. A regular documentation of stimulation and sensory parameters allows individually a close-mesh optimisation of occipital nerve stimulation treatment in future. Through the internet-based application, the tool can be used internationally and multi-centrically.

No conflict of interest.

